# Ecology and Host Identity Outweigh Evolutionary History in Shaping the Bat Microbiome

**DOI:** 10.1128/mSystems.00511-19

**Published:** 2019-11-12

**Authors:** Holly L. Lutz, Elliot W. Jackson, Paul W. Webala, Waswa S. Babyesiza, Julian C. Kerbis Peterhans, Terrence C. Demos, Bruce D. Patterson, Jack A. Gilbert

**Affiliations:** a Integrative Research Center, Field Museum of Natural History, Chicago, Illinois, USA; b Department of Pediatrics, University of California San Diego, La Jolla, California, USA; c Scripps Institution for Oceanography, University of California San Diego, La Jolla, California, USA; d Department of Microbiology, Cornell University, Ithaca, New York, USA; e Department of Forestry and Wildlife Management, Maasai Mara University, Narok, Kenya; f Department of Wildlife Management, Sokoine University of Agriculture, Morogoro, Tanzania; g Department of Biological Sciences, Roosevelt University, Chicago, Illinois, USA; Australian Institute of Marine Science

**Keywords:** microbiome, Chiroptera, phylosymbiosis, Afrotropics

## Abstract

Recent studies of mammalian microbiomes have identified strong phylogenetic effects on bacterial community composition. Bats (Mammalia: Chiroptera) are among the most speciose mammals on the planet and the only mammal capable of true flight. We examined 1,236 16S rRNA amplicon libraries of the gut, oral, and skin microbiota from 497 Afrotropical bats (representing 9 families, 20 genera, and 31 species) to assess the extent to which host ecology and phylogeny predict microbial community similarity in bats. In contrast to recent studies of host-microbe associations in other mammals, we found no correlation between chiropteran phylogeny and bacterial community dissimilarity across the three anatomical sites sampled. For all anatomical sites, we found host species identity and geographic locality to be strong predictors of microbial community composition and observed a positive correlation between elevation and bacterial richness. Last, we identified significantly different bacterial associations within the gut microbiota of insectivorous and frugivorous bats. We conclude that the gut, oral, and skin microbiota of bats are shaped predominantly by ecological factors and do not exhibit the same degree of phylosymbiosis observed in other mammals.

**IMPORTANCE** This study is the first to provide a comprehensive survey of bacterial symbionts from multiple anatomical sites across a broad taxonomic range of Afrotropical bats, demonstrating significant associations between the bat microbiome and anatomical site, geographic locality, and host identity—but not evolutionary history. This study provides a framework for future systems biology approaches to examine host-symbiont relationships across broad taxonomic scales, emphasizing the need to elucidate the interplay between host ecology and evolutionary history in shaping the microbiome of different anatomical sites.

## Abstract (precis)

This study is the first to provide a comprehensive survey of bacterial symbionts from multiple anatomical sites across a broad taxonomic range of Afrotropical bats, demonstrating significant associations between the bat microbiome and anatomical site, geographic locality, and host identity—but not evolutionary history. This study provides a framework for future systems biology approaches to examine host-symbiont relationships across broad taxonomic scales, emphasizing the need to elucidate the interplay between host ecology and evolutionary history in shaping the microbiome of different anatomical sites.

## INTRODUCTION

Animals rely on bacterial symbionts for many basic biological functions, such as digestion and immune system development (1, 2). Many studies have found significant associations between host phylogeny (shared ancestry) and bacterial community composition (3–6), while others have identified dietary or spatiotemporal variables as significant drivers of host-microbe associations over the course of an individual life span (7–10). This variation may reflect the extent to which hosts depend on their associated microbes for key functions. Host-microbe relationships that are conserved over evolutionary time may indicate a significant functional role of microbes in their hosts, while microbial associations that are not conserved across host phylogeny may represent incidental or transient host-microbe relationships. Furthermore, the influence of microbes on their hosts may be context dependent, such that the presence of a particular microbe may be beneficial under one set of ecological conditions and harmful under another (11, 12), leading to stochastic patterns of microbial associations in different host lineages.

The occurrence of closely related hosts sharing more similar microbiota than distantly related hosts—a pattern termed “phylosymbiosis”—has been observed among many animal groups (6, 13–16) and across evolutionary timescales spanning millions of years (6, 17, 18). This process-independent pattern may result from coevolution between hosts and microbial symbionts, neutral population dynamics, or ecological filtering driven by specific host traits such as diet, age, sex, and body size (19). Many host traits are phylogenetically conserved, confounding the extent to which ecological versus evolutionary factors contribute to phylosymbiosis. The strength of phylosymbiosis may also be influenced by organ-specific exposure to the environment (1). Although the majority of studies in which phylosymbiosis is identified have focused on microbes inhabiting internal organs, such as the gastrointestinal tract, recent work suggests that external host environments (e.g., skin) can also exhibit a signal of phylosymbiosis (20). However, comparisons across internal and external sites within the same individuals or host species have rarely been explored.

Bats (Mammalia: Chiroptera) are a unique system for comparison of the relative contributions of evolutionary and ecological factors driving host-symbiont associations. Bats are among the most speciose orders of mammals (second only to the order Rodentia) and exhibit a wide range of dietary niches (21–23). Flight allows bats to access wide geographic and ecological ranges relative to their nonflighted mammalian counterparts (24–26), and it has led to the evolution of unique metabolic and physiological adaptations (27). Interestingly, despite the widespread, consistent observance of phylosymbiosis among mammals (3, 6, 20, 28–31), bats appear to be an exception, with recent studies presenting conflicting results on the role of bat phylogeny in shaping the microbiome (3, 32–34). To date, most studies of bat microbiota have included only a small number of wild species or captive individuals, and among bat gut microbiome studies, most have focused primarily on the Neotropical family Phylostomidae.

In this study, we conducted the first broad-scale analysis of Afrotropical bat-associated microbes from 9 families and 20 genera to determine the extent to which bacterial communities among bats follow a pattern of phylosymbiosis or stochastic assembly. We concurrently sampled the distal colon (gut hereafter), skin, and oral cavities of each individual to measure associations between bacterial community composition across anatomical sites within and between hosts. Last, we reconstruct host phylogeny to explicitly test for the significance of phylosymbiosis between bats and their gut, oral, and skin microbiota, while assessing the effects of host-specific traits, including diet, age, sex, and mass, and ecological features, including locality and elevation on bacterial community composition. On the basis of the results of these analyses, we conclude that bats are unique among mammals, showing limited evidence for phylosymbiosis with bacterial symbionts while maintaining strong taxonomic and ecological effects across all three anatomical sites.

## RESULTS

### Microbial richness associated with bat skin is significantly greater than gut or oral microbial communities.

**FIG 1:**
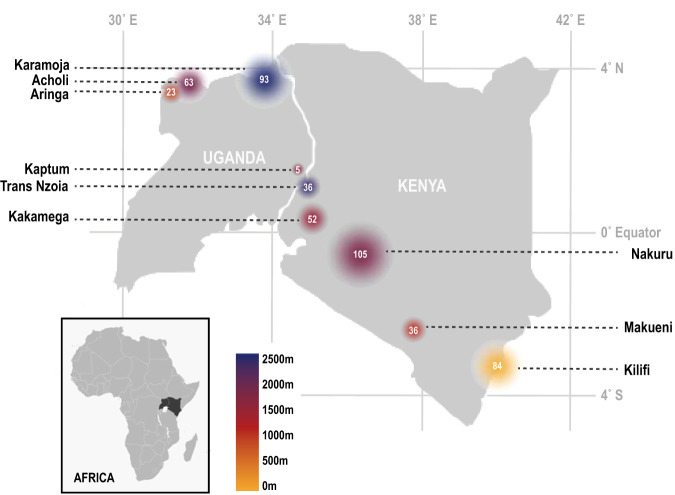
Sampling localities and elevation, grouped by district (see Table S1 in the supplemental material for full locality information). Colors correspond to elevation, and white numbers and the size of circles correspond to the number of bats collected.

Sampling was conducted across 20 sites in Kenya and Uganda from July-August of 2016, ranging from sea level to ∼2,500 m in elevation (Fig. 1; see Table S1 in the supplemental material). We collected gut, oral, and skin samples for bacterial community characterization from a total of 497 individual bats, comprising 9 families, 20 genera, and 31 species. An average of four bat families were sampled per district (see Tables S2 and S3 for full host metadata and summary of host species sampling per site, respectively). All host vouchers are accessioned at the Field Museum of Natural History (Chicago, IL, USA) (Table S2).

10.1128/mSystems.00511-19.6 TABLE S1 Full sampling locality data. The county name corresponds to map labels in Fig. 1 of the main text of this article. Download Table S1, XLSX file, 0.04 MB. Copyright © 2019 Lutz et al. 2019 Lutz et al. This content is distributed under the terms of the Creative Commons Attribution 4.0 International license.

10.1128/mSystems.00511-19.7 TABLE S2 Host voucher data and sampling. Download Table S2, XLSX file, 0.2 MB. Copyright © 2019 Lutz et al. 2019 Lutz et al. This content is distributed under the terms of the Creative Commons Attribution 4.0 International license.

10.1128/mSystems.00511-19.8 TABLE S3 Host counts by site. Download Table S3, XLSX file, 0.04 MB. Copyright © 2019 Lutz et al. 2019 Lutz et al. This content is distributed under the terms of the Creative Commons Attribution 4.0 International license.

**FIG 2:**
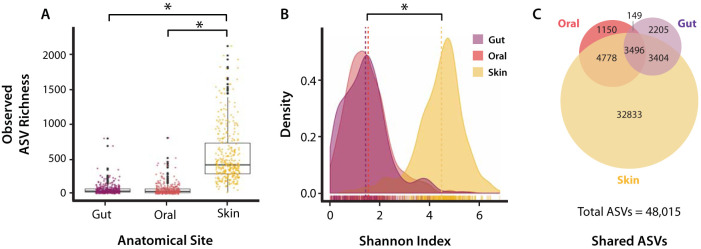
α-Diversity of amplicon sequence variants (ASVs) by anatomical sites, including observed ASV richness (A), Shannon index of diversity (B), ASVs shared between anatomical sites (C). Asterisks indicate significant differences between groups (Dunn’s test, Bonferroni-corrected *P* < 0.0001).

Following sample processing, sequencing, and quality control, a total of 1,236 16S rRNA amplicon libraries were generated, with an average read depth of 32,950 reads per library (standard deviation [SD], ±19,850 reads). A total of 1,111 libraries were retained following further quality filtering and included 368 libraries for gut samples, 343 libraries for oral samples, and 400 libraries for skin samples (Table S1). Across all samples, 48,015 amplicon sequence variants (ASVs) were identified using Deblur (35). Gut microbial communities exhibited the lowest overall richness (9,254 ASVs), followed closely by oral microbial communities (9,573 ASVs), while the microbial richness of skin microbial communities (44,511 ASVs) was significantly greater than that of gut or oral (*P* < 2.2e−16, Kruskal-Wallis rank sum test; Bonferroni-corrected *P* value *P* < 1e−113, Dunn’s test) (Fig. 2; see also Fig. S1 in the supplemental material). The mean observed ASVs by anatomical site were 78.6 (SD, ±55.5), 104.3 (SD, ±53.3), and 633.7 (SD, ±274.5) for gut, oral, and skin samples, respectively (Table S4). The Shannon index score of skin microbial communities was also significantly greater than that of gut or oral microbial communities (*P* < 2.2e−16, Kruskal-Wallis test; Bonferroni-corrected *P* value *P* < 1e−119, Dunn’s test). We identified 3,496 ASVs that were shared by all three body sites, while there were 6,900 ASVs shared between the oral and skin microbiota, 8,274 ASVs shared between gut and skin microbiota, and 3,645 ASVs shared between oral and gut microbiota (Fig. 2; Fig. S1). Analysis of α-diversity across geographic localities by elevation revealed that bats at higher elevations tended to exhibit increased Shannon index (SI) of diversity and observed richness (OR) across oral microbiota (for SI, *R*^2^ = 0.076, *P* < 3.1e−9; for OR, *R*^2^ = 0.038, *P* < 2.5e−5) and skin (for SI, *R*^2^ = 0.16, *P* < 2.2e−16; for OR, *R*^2^ = 0.100, *P* < 2.5e−14) microbiota, while only observed richness of the gut was significantly correlated with elevation (for OR, *R*^2^ = 0.01, *P* < 0.015) (Fig. S2).

10.1128/mSystems.00511-19.1 FIG S1 α-Diversity measures of data rarefied to a read depth of 10,000. (A) Observed ASV richness across anatomical sites. Gut and oral microbial richness differed significantly from skin microbial richness (Kruskal-Wallis chi-squared = 677.01, df = 2, *P* < 2.2e−16, Dunn’s test), but did not differ significantly from each other. (B) Density plots of Shannon index (SI) by anatomical site. SI of skin microbial richness and evenness differed significantly from the SI of both gut and oral microbiomes, which did not differ from each other (Kruskal-Wallis chi-squared = 678.0885, df = 2, *P* < 2.2e−16, Dunn’s test). (C) Venn diagram of shared and specific ASVs across different anatomical sites. As with analyses of the nonrarefied data set, the skin microbiome exhibited the highest diversity (27,825 ASVs), followed by the oral microbiome (10,696 ASVs), and last, the gut microbiome (6,492 ASVs). Rarefying data led to a loss of 1,079 ASVs (4% of total ASVs) that did not appear in any sample after rarefaction, and the removal of 1,079 libraries that had <10,000 reads. (D) Mean ASV richness as read depth increases, with removal of libraries containing fewer than the identified number of reads (note that the color key in panel D differs from the color key in panels A to C). Download FIG S1, PDF file, 1.0 MB. Copyright © 2019 Lutz et al. 2019 Lutz et al. This content is distributed under the terms of the Creative Commons Attribution 4.0 International license.

10.1128/mSystems.00511-19.2 FIG S2 Linear regression of Shannon diversity (A) and observed ASV richness (B) of gut, oral, and skin microbiomes against elevation from which host was sampled (∼0 to 2,500 m above sea level). *R*^2^ and significance values are provided within each plot; shading corresponds to 95% confidence interval. Download FIG S2, PDF file, 1.1 MB. Copyright © 2019 Lutz et al. 2019 Lutz et al. This content is distributed under the terms of the Creative Commons Attribution 4.0 International license.

10.1128/mSystems.00511-19.9 TABLE S4 Average individual α-diversity of microbial communities across anatomical sites within each host genus, measured by Shannon index of diversity (SI) and observed ASV richness (Obs) with standard deviation (SD). *n* is the number of libraries included in each calculation following quality filtering. Download Table S4, XLSX file, 0.1 MB. Copyright © 2019 Lutz et al. 2019 Lutz et al. This content is distributed under the terms of the Creative Commons Attribution 4.0 International license.

### Microbial communities significantly correlate with host taxonomy and ecological variables, but not host phylogeny.

Permutational multivariate analysis of variance (PERMANOVA) analyses of unweighted (UF) and weighted UniFrac (WUF) microbial β-diversity identified a number of variables as significant factors explaining variation among all three anatomical sites, including host taxon (at the family, genus, and species level) (Table 1), geographic locality, diet, mass, and age (Table 2). Across all three anatomical sites, the effect of host taxonomy diminished with increasing taxonomic level (e.g., *R*^2^ of host species > *R*^2^ of host genus > *R*^2^ of host family), and host species consistently explained the most variation for the gut microbiota (for UF, *R*^2^ = 0.19, *P* = 0.001; for WUF, *R*^2^ = 0.24, *P* = 0.001), oral microbiota (for UF, *R*^2^ = 0.24, *P* = 0.001; for WUF, *R*^2^ = 0.49, *P* = 0.001), and skin microbiota (for UF, *R*^2^ = 0.24, *P* = 0.001; for WUF, *R*^2^ = 0.40, *P* = 0.001) microbiota. Geographic locality explained the second highest level of variation following host taxonomy, and in fact, it explained more variation than host species for the skin microbiome (for UF, *R*^2^ = 0.11, *P* = 0.001; for WUF, *R*^2^ = 0.18, *P* = 0.001), while explaining 5 to 10% of marginal variation in the gut microbiota (for UF, *R*^2^ = 0.09, *P* = 0.001; for WUF, *R*^2^ = 0.07, *P* = 0.001) and oral microbiota (for UF, *R*^2^ = 0.08, *P* = 0.001; for WUF, *R*^2^ = 0.05, *P* = 0.001) (Table 1). The results of analyses based on reduced data sets (host species for which sample sizes were *N* ≥ 10 and *N* ≥ 20) did not differ substantially from analyses performed on the entire data set, with the exception of age and sex no longer found to be significant in the *N* ≥ 20 data set for unweighted UniFrac metrics of the oral microbiome and age no longer found to be significant in the *N* ≥ 20 data set for the weighted UniFrac metrics of the oral microbiome. We also found that sex became a significant predictor for weighted UniFrac dissimilarity of the oral microbiome among the *N* ≥ 20 data set. No significant differences were observed between other anatomical sites, host traits, or host taxonomic levels, and no differences were observed for the *N* ≥ 10 data set analyses (Table S5).

**TABLE 1:** Permutational multivariate analysis of variance by nested taxonomic variables based on unweighted and weighted UniFrac distance metrics^*a*^

Site	Host taxonomic level	Unweighted UniFrac				Weighted UniFrac			
		Sum sqrs	*R*^2^	*F*	Pr(>*F*)	Sum sqrs	*R*^2^	*F*	Pr(>*F*)
Gut	Family	6.35	0.08	3.41	0.001	2.63	0.10	4.66	0.001
	Family(genus)	10.8	0.13	2.82	0.001	4.97	0.19	4.49	0.001
	Family(genus(species))	15.85	0.19	2.95	0.001	6.39	0.24	4.1	0.001

Oral	Family	10.05	0.11	4.78	0.001	4.41	0.30	16.43	0.001
	Family(genus)	15.47	0.17	3.6	0.001	5.12	0.35	9.37	0.001
	Family(genus(species))	21.56	0.24	3.33	0.001	7.18	0.49	10.2	0.001
									
Skin	Family	11.93	0.12	6.12	0.001	4.11	0.20	11.11	0.001
	Family(genus)	16.32	0.16	4.31	0.001	5.44	0.26	7.8	0.001
	Family(genus(species))	23.84	0.24	3.97	0.001	8.27	0.40	8.37	0.001

a For unweighted and weighted UniFrac distance metrics, the sum of squares (Sum sqrs), *R*^2^, *F*, and probability of >*F* [Pr(>*F*)] are shown.

**TABLE 2:** Permutational multivariate analysis of variance of host variables based on unweighted and weighted UniFrac distance metrics

Site	Formula	Variable	Unweighted UniFrac				Weighted UniFrac			
			Sum sqrs	*R*^2^	*F*	Pr(>*F*)	Sum sqrs	*R*^2^	*F*	Pr(>*F*)
Gut	1^*a*^	Host species	10.03	0.12	2.07	0.001	4.12	0.16	2.90	0.001
		Locality	7.35	0.09	2.43	0.001	1.9	0.07	2.14	0.001

	2^*b*^	Diet	2.14	0.03	8.93	0.001	1.12	0.04	15.28	0.001

	3^*c*^	Mass	1.78	0.02	7.43	0.003	0.96	0.04	13.08	0.003
		Age	0.75	0.01	1.57	0.011	0.27	0.01	1.82	0.06
		Sex	0.46	0.01	1.92	0.009	0.04	<0.01	0.58	0.86

Oral	1	Host species	12.14	0.13	2.17	0.001	4.21	0.29	6.80	0.001
		Locality	7.62	0.08	2.62	0.001	0.7	0.05	2.18	0.001

	2	Diet	1.93	0.02	6.81	0.001	2.09	0.14	51.98	0.001

	3	Mass	1.71	0.02	6.12	0.002	1.3	0.09	31.73	0.003
		Age	1.51	0.02	2.69	0.002	0.25	0.02	3.08	0.006
		Sex	0.31	<0.01	1.09	0.293	0.14	0.01	3.38	0.01

Skin	1	Locality	11.3	0.11	3.8	0.001	3.76	0.18	9.41	0.001
		Host species	10.1	0.10	1.96	0.001	3.13	0.15	4.53	0.001

	2	Diet	5.28	0.05	20.52	0.001	1.14	0.06	21.18	0.001

	3	Mass	3.32	0.03	13	0.002	0.75	0.04	14.20	0.002
		Age	2.05	0.02	4.02	0.002	0.43	0.02	4.07	0.002
		Sex	0.34	<0.01	1.31	0.148	0.07	<0.01	1.38	0.161

a Formula 1. adonis2([data] ∼ Locality + Host species, by = “margin,” perm = 999).

b Formula 2. adonis2([data] ∼ Diet, strata = Locality, perm = 999).

c Formula 3. adonis2([data] ∼ Age + Sex + Mass, strata = Host species, by = “margin,” perm = 999).

10.1128/mSystems.00511-19.10 TABLE S5 PERMANOVA tests of host traits and host taxon with nesting using unweighted and weighted UniFrac β-diversity metrics estimated for data sets reduced to include only (i) species for which >10 individuals were sampled and (ii) species for which >20 individuals were sampled. Download Table S5, XLSX file, 0.1 MB. Copyright © 2019 Lutz et al. 2019 Lutz et al. This content is distributed under the terms of the Creative Commons Attribution 4.0 International license.

Although significant, diet explained ≤5% of variation among gut microbiota (for UF, *R*^2^ = 0.03, *P* = 0.001; for WUF, *R*^2^ = 0.04, *P* = 0.001) and skin microbiota (for UF, *R*^2^ = 0.05, *P* = 0.002; for WUF, *R*^2^ = 0.06, *P* = 0.002). Interestingly, diet explained up to 14% of variation among oral microbiota (for UF, *R*^2^ = 0.02, *P* = 0.001; for WUF, *R*^2^ = 0.14, *P* = 0.001). We note that in assessing beta dispersion of our grouping variables, we rejected the null hypothesis that host species and locality had the same within-group dispersion values for gut, oral, and skin microbiota, which may affect PERMANOVA outcomes (Fig. S3 and S4); for the category of diet, beta dispersion did not vary significantly between the two dietary groups with respect to gut or skin microbiota, but it did vary significantly for oral microbiota (Fig. S5). We also note that although sampling effort (i.e., sample sizes) varied across host species and sampling sites, additional PERMANOVA analyses performed on two data subsets including only host species for which *N* ≥ 10 and *N* ≥ 20 individuals were sampled did not produce meaningfully different results (Table S5).

10.1128/mSystems.00511-19.3 FIG S3 Weighted UniFrac beta dispersion (A) and principal coordinate analysis (PCoA) (B) plots of weighted UniFrac distances across sampling localities and anatomical sites. Ellipses correspond to 95% confidence level per locality grouping. Download FIG S3, PDF file, 0.9 MB. Copyright © 2019 Lutz et al. 2019 Lutz et al. This content is distributed under the terms of the Creative Commons Attribution 4.0 International license.

10.1128/mSystems.00511-19.4 FIG S4 Weighted UniFrac beta dispersion (A) and PCoA (B) plots of weighted UniFrac distances within bat species and anatomical sites. Ellipses correspond to 95% confidence level per host species grouping. Download FIG S4, PDF file, 0.9 MB. Copyright © 2019 Lutz et al. 2019 Lutz et al. This content is distributed under the terms of the Creative Commons Attribution 4.0 International license.

10.1128/mSystems.00511-19.5 FIG S5 Analyses of weighted UniFrac beta dispersion for gut, oral, and skin microbiomes. (A) Distance of samples from group centroid, with analysis of variance (ANOVA) results shown in the top right corners. (B) PCoA plots of gut, oral, and skin microbiota. Data for frugivorous bats (light blue) and insectivorous bats (dark blue) are indicated. Download FIG S5, PDF file, 1.3 MB. Copyright © 2019 Lutz et al. 2019 Lutz et al. This content is distributed under the terms of the Creative Commons Attribution 4.0 International license.

**FIG 3:**
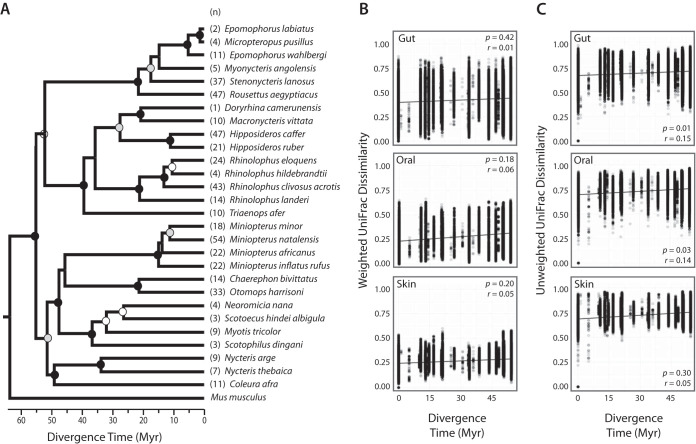
Ultrametric BEAST chronogram (A) and rate of microbiome divergence across phylogenetic distance of bats (B and C). Strengths of correlations assessed by Mantel tests (10,000 permutations) of pairwise divergence estimates of host species and weighted UniFrac microbial community dissimilarity (B) and unweighted UniFrac microbial community dissimilarity (C) are shown. Divergence time is shown in million years (Myr). The *P* values and Mantel *r* statistics are shown in the top or bottom right corners of the plots. Circles at nodes correspond to Bayesian posterior probability of >99% (black), >90% (gray), and >80% (white).

We reconstructed an ultrametric host phylogeny using DNA from bats collected during this study or otherwise accessioned at the Field Museum of Natural History; the resulting topology was well supported at most major nodes, and compared to that of Amador et al. (36) found to be largely congruent with the exception of relationships between Molossidae, Miniopteridae, and Vespertilionidae (see Materials and Methods). Mantel tests of host phylogenetic distances and weighted UniFrac microbial community dissimilarity values revealed no correlation between gut (*P* = 0.42, *r* = 0.01), oral (*P* = 0.18, *r* = 0.06), or skin (*P* = 0.20, *r* = 0.05) microbiota and host phylogenetic distance (Fig. 3). Mantel tests based on unweighted UniFrac dissimilarity values found significant but weak correlations between host phylogeny and both gut microbial dissimilarity (*P* = 0.014, *r* = 0.15) and oral microbial dissimilarity (*P* = 0.03, *r* = 0.14), but not between skin microbial dissimilarity and host phylogeny (Fig. 3). Additional Mantel tests performed on host phylogenetic distances estimated from a nonultrametric tree did not produce meaningfully different results, with the exception that gut and oral unweighted UniFrac dissimilarities no longer significantly correlated with host phylogeny.

### Differences in microbial community composition between bat taxa, anatomical sites, and dietary niches.

**FIG 4:**
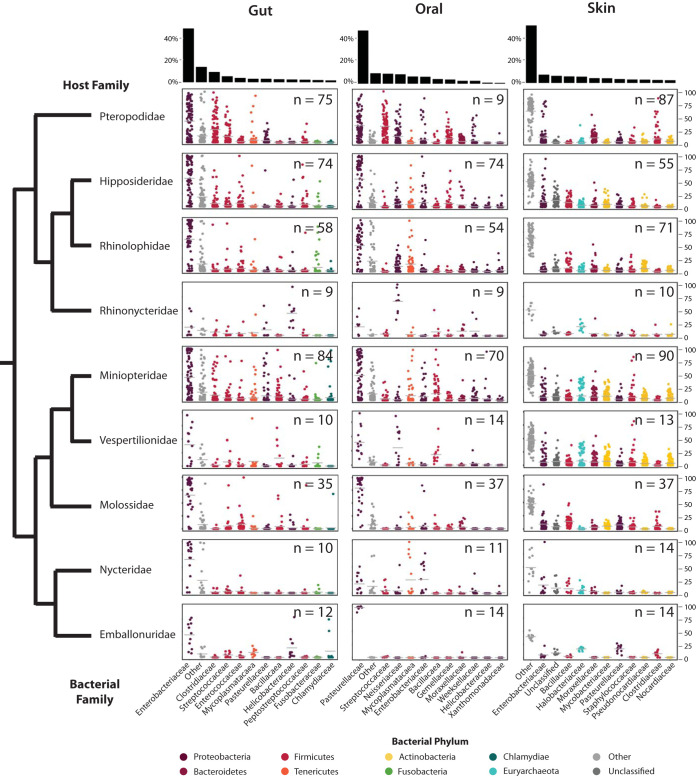
Percent relative abundance of top 11 bacterial families identified in gut, oral, and skin microbiomes of bats. Points correspond to individual libraries. Bacterial families are colored according to bacterial phylum. The number of libraries is indicated in the top right-hand corner of each plot. The black bar graphs at the top of the figure indicate average relative abundances.

Across all bat species, the gut microbiome was dominated by *Proteobacteria* (mean ± standard error [SE], 60.4% ± 1.6%), which comprised mainly *Enterobacteriaceae* (50.0% ± 1.8%). *Firmicutes* were also highly abundant (27.4% ± 1.4%), comprising mainly *Clostridiaceae* (9.5% ± 1.0%) and *Streptococcaceae* (5.5% ± 0.6%). Oral microbiota were dominated by *Proteobacteria* (68.0% ± 1.4%) comprising families differing from those found in the gut, including *Pasteurellaceae* (47.5% ± 1.8%) and *Neisseriaceae* (8.3% ± 0.8%), among other bacterial families of lower average abundance (Fig. 4). *Firmicutes* also were somewhat abundant (18.5% ± 1.1%), comprising *Streptococcaceae* (8.8% ± 0.8%) and *Gemellaceae* (3.6% ± 0.4%). The skin microbiome was not dominated by one particular bacterial phylum, but it did exhibit high abundances of *Proteobacteria* (35.3% ± 0.8%) and *Actinobacteria* (23.0% ± 0.6%). *Proteobacteria* were primarily comprised by *Enterobacteriaceae* (7.4% ± 0.7%); *Actinobacteria* were primarily comprised by *Mycobacteriaceae* (4.1% ± 0.4%), *Pseudonocardiaceae* (2.8% ± 0.2%), and *Nocardiaceae* (2.3% ± 0.2%). *Bacteroidetes* (*Moraxellaceae*, average [avg] 5.6%) and *Euryarchaeota* (*Halobacteriaceae*, avg 4.2%) comprised the next most abundant bacterial groups across the skin microbiota of bats. Despite these general trends, the relative abundance of specific bacteria varied both within and between host families (Fig. 4).

Supervised machine learning analyses (random forests) produced models that could classify the host genus, species, locality, and diet of oral, and skin microbial samples with a ratio of baseline to observed classification error of >2. Random forest performance was assessed by confusion matrix outputs (i.e., percentage of properly classified to improperly classified variable states) and by comparing the out-of-bag estimated error (OOB) with baseline (random) error. If the ratio of OOB to baseline error was greater than or equal to 2, the model was considered to perform reasonably well, as it performed at least twice as well as random (37). Accuracy of classification models was highest for skin microbiota and host diet, followed by skin and host locality. Random forest models were also able to classify microbial communities of the gut into accurate dietary groups (frugivore and insectivore), and to a lesser extent were able to classify gut microbiota by host genus, species, and locality (Table 3). Of all random forest analyses, the oral microbiome emerged as the strongest predictor of host diet.

**TABLE 3:** Random forest classification of microbiota by host variables

Classifier	Anatomical site	Classification error^*a*^ (%) (avg ± SD)	Baseline/OOB^*b*^
Host family	Gut	52 ± 35	3.74
	Oral	16 ± 20	7.69
	Skin	40 ± 43	5.01

Host genus	Gut	68 ± 36	3.05
	Oral	45 ± 45	5.69
	Skin	59 ± 45	4.56

Host species	Gut	65 ± 36	2.64
	Oral	53 ± 40	3.64
	Skin	56 ± 42	3.86

Host locality	Gut	65 ± 32	2.95
	Oral	56 ± 35	3.61
	Skin	35 ± 37	9.01

Host diet	Gut	13 ± 16	3.95
	Oral	4 ± 02	6.73
	Skin	4 ± 05	9.67

a Classification error is estimated as the ratio of incorrect classification occurrence to total classifications; values closer to 0% indicate lower error rate.

b The ratio of baseline error to out-of-bag (OOB) estimated error with 1,000 bootstrap replicates is shown.

**FIG 5:**
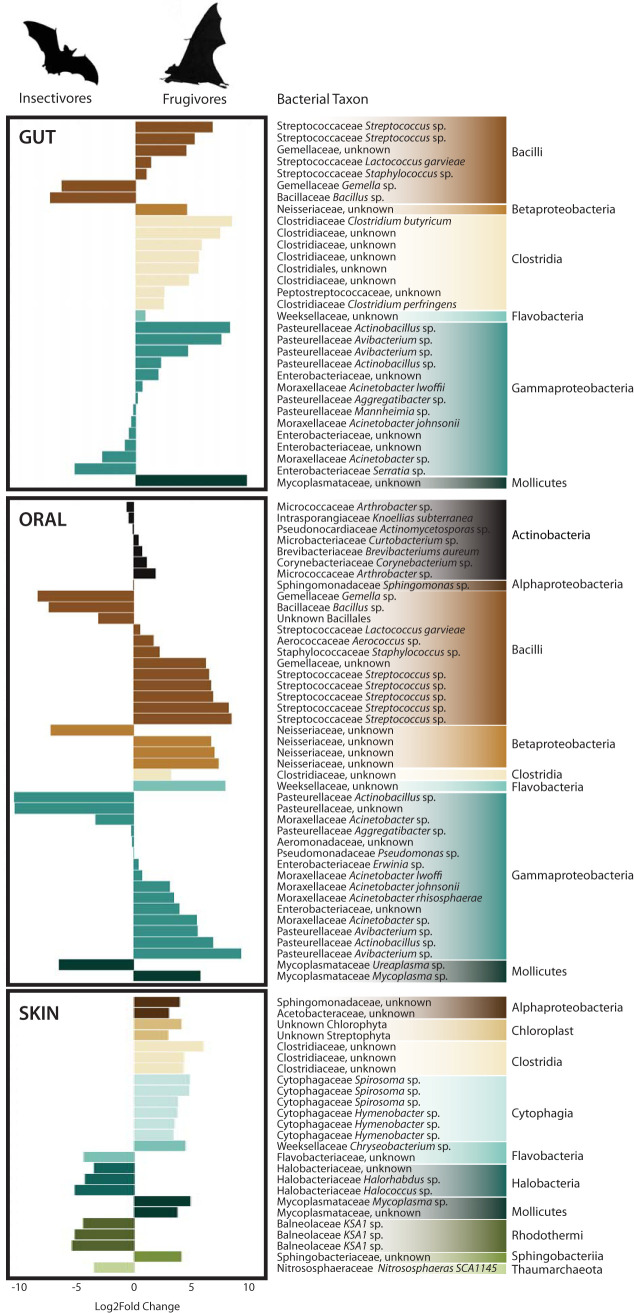
Analysis of composition of microbiomes (ANCOM) identified ASVs that were differentially abundant within the gut, oral, and skin microbiomes of insectivorous and frugivorous bats. Bars correspond to the magnitude of the log_2_ fold change in relative abundance of each ASV, with negative values associated with an increase among insectivores and positive values associated with an increase among frugivores. ASVs identified at the 70% detection threshold for gut and oral microbiomes and at the 90% detection threshold for skin are shown.

Analysis of the composition of microbiomes (ANCOM) among gut microbiota identified individual bacterial ASVs that were differentially associated with frugivorous versus insectivorous bats (Fig. 5). Among the gut microbial community, ASVs belonging to the families *Clostridiaceae*, *Pasteurellaceae*, *Streptococcaceae*, and *Mycoplasmataceae* were significantly associated with fruit bats, and ASVs belonging to the *Bacillaceae* and *Enterobacteriaceae* were significantly associated with insectivorous bats. Many of the differences found within the gut are also observed in the oral microbial communities with the exception of *Neisseriaceae* largely being associated with fruit bats. Among the skin microbial community, ASVs mostly of *Halobacteriaceae* and *Balneolaceae* were significantly associated with insectivorous bats, while ASVs of *Cytophagaceae* and *Clostridiaceae* among others were significantly associated with fruit bats.

## DISCUSSION

In this study, we surveyed the microbiome of a broad range of Afrotropical bats from multiple anatomical sites and analyzed the effects of host taxonomy, phylogeny, and ecological factors on shaping the bat microbiome. The bacterial diversity we observed among gut, oral, and skin microbiota of bats falls within ranges observed in other vertebrate groups (3, 38–41). We found the skin microbiome to be consistently higher in both α- and β-diversity measures relative to the gut and oral microbiota. This result is consistent with other mammalian microbiome studies reporting significantly higher bacterial diversity on the skin compared to other anatomical regions (42–44). Host taxonomy explained the greatest amount of variation among microbial β-diversity, and its effect decreased with increasing taxonomic rank, i.e., host species explained greater variation of β-diversity than host genus or host family. Similar to studies of North American bats (34), we found sampling locality to be a significant factor influencing skin, gut, and oral microbial composition. As shown in other North American mammals by Moeller et al. (45), isolation by distance of bacterial communities may act to accelerate β-diversity shifts in the gut microbiome. Although some bat species are known to migrate locally and over long distances (46–48), the movement ecology of many species in this study is poorly known. The effect of sampling locality that we observed suggests that the movement of bats in our study group and/or during our sampling period may be fairly localized, though more research is needed in this area. We also observed a trend of increasing Shannon diversity and observed ASV richness along an elevational gradient that was most pronounced for skin microbiota. A positive correlation between bacterial richness and elevation has been observed in studies of amphibian skin (49) and montane soil, and this pattern may be the result of climatological and other abiotic factors (e.g., pH) found along elevational gradients (50, 51).

The general composition of gut microbiota in bats is strikingly different from the composition of other mammalian gut microbiomes, which are generally dominated by *Firmicutes*. Interestingly, the high relative abundance of *Proteobacteria* in the chiropteran gut is similar to that found in the avian gut, suggesting the possibility of convergence with respect to the microbiome in flighted vertebrates (52). Regardless of diet, the distal bat gut is dominated by bacteria in the family *Enterobacteriaceae* (phylum *Proteobacteria*), though fruit bats hosted an increased relative abundance of *Clostridiaceae* (phylum *Firmicutes*) and *Streptococcaceae* (phylum *Firmicutes*) compared to insectivorous bats, a finding previously observed in Neotropical bats (33). Many bacterial species in the *Lactobacillales* are known associates of fermenting fruit (53, 54); thus, the presence of *Streptococcaceae* (order *Lactobacillales*) in the fruit bat gut may be due to ingestion rather than established residence. Whether these bacteria are resident or transient requires further investigation.

Unlike the gut bacterial community, the oral microbiome of bats was broadly found to be more similar to other mammalian species. The bat oral microbiome was dominated by *Pasteurellaceae* (phylum *Proteobacteria*), and in some cases, a high relative abundance of bacteria in the families *Mycoplasmataceae* (in nycterids), *Neisseriaceae* (in vespertilionids and rhinonycterids), and *Streptococcaceae* (in pteropodids) was also observed. Although the oral microbiome has received limited attention relative to the gut, several studies have found diverse *Pasteurellaceae* and *Neisseria* lineages present in the oral microbiota of animals, including domestic cats (38), marine mammals (55), and Tasmanian devils (41, 56). In humans, *Pasteurellaceae* (genera *Haemophilus* and *Aggregatibacter*) and *Neisseriaceae* (genera *Neisseria*, *Kingella*, and *Eikenella*) play an important role in the formation of supragingival plaque (40). Though these bacterial groups are present in lower proportions in other animals compared to bats, their presence in the oral microbiome of a broad range of vertebrate taxa suggests that they may serve an important function in the oral microbiome of vertebrates.

Although we observed a strong species-level effect on bacterial β-diversity, we found no significant correlation between chiropteran phylogeny and weighted UniFrac bacterial dissimilarities and found significant but weak correlations between host phylogeny and unweighted UniFrac dissimilarities. These results corroborate the findings of Nishida and Ochman (3) and suggest that species-level effects are driven by shared ecological features rather than host phylogeny. The significant but weak correlations between host phylogenetic distance and gut and oral unweighted UniFrac microbial dissimilarity suggest that perhaps a small number of bacterial taxa are evolutionarily conserved in their associations with bats but that these taxa are in relatively low abundance and perhaps limited to internal organs, which are less exposed to the external environment (relative to skin, for example). Our findings differ markedly from studies of all other mammals, among which phylogenetic relatedness is generally significantly correlated with microbial community dissimilarity (16, 29, 39, 45). Strong species-level effects on the gut microbiome have also been observed in other mammals and have been attributed to differences in dietary preference, gut physiology, or other species-specific features (57, 58). Interspecific variation outweighing intraspecific variation has been suggested as evidence for phylosymbiosis, and phylosymbiosis is indeed observed in many host systems with a higher ratio of inter- to intraspecific microbiome variation. Although this pattern is a key feature of phylosymbiosis, it alone is not sufficient to conclude that closely related host species share more similar microbiomes due to phylogeny, as the pattern may be driven by shared ecology of closely related species rather than host evolution (6). This can be seen in studies where despite a strong species-level effect on microbiome composition, microbial community does not recapitulate host phylogeny (52). Conversely, several recent studies have suggested that phylosymbiosis is observed in bats based on the recapitulation of host phylogeny by microbial community dendrograms and significant species-level effects (32, 33, 59). Such studies, however, have tended to incorporate only a small number of taxa that are often closely related or do not incorporate explicit tests of host phylogenetic distance and microbial community dissimilarity for tests of phylosymbiosis. They are therefore limited in their ability to disentangle the effects on the microbiome that are due to host ecology versus phylogeny, and indeed, find strong effects of host ecology on the microbiome as well.

As suggested in other studies of flighted vertebrates (bats and birds), convergent adaptations driven by the evolution of flight may influence digestive physiology, such as increasing paracellular absorption and accelerating the transit time of food through the gut (60–63). These physiological adaptations to flight may in turn affect the nature and composition of microbial communities in flighted vertebrates, providing one possible explanation for the absence of a phylogenetic correlation between bats and their microbes. A recent study of 59 Neotropical bird species found little congruence between the topologies of microbial and host phylogenies, despite finding a significant effect of host species on microbiome composition (52). Flight is an energetically demanding form of locomotion that requires the animal to have a digestive system that meets its high metabolic needs with a gut that is low in weight. Relative to most nonflighted mammals, bats have comparatively smaller gastrointestinal (GI) tracts, reduced intestinal tissue, and smaller digestive loads that help to minimize flight mass (60, 62, 64, 65). Bats also have relatively streamlined GI tract design, regardless of dietary niche, that lacks a cecum and appendix (66), but still achieves high digestive efficiencies (65, 67). This phenomenon is potentially explained by the ability of small birds and bats to meet high metabolic demands via paracellular nutrient absorption (61). We hypothesize that the energetic demands of flight place significant constraints on gut physiology that in turn restrict the microbial community found in the gut, resulting in the observed absence of correlation between host phylogeny and weighted UniFrac microbiome composition. Given the significant but weak correlation between host phylogeny and unweighted UniFrac dissimilarities of gut and oral microbiomes, additional studies that can identify low-abundance bacterial taxa that are conserved across the phylogeny may shed light on their functional significance.

Our comparative analysis of three anatomical sites across 31 bat species from 20 sites in East Africa revealed a strong effect of host taxon, geographic locality, and to a lesser extent diet and elevation on microbiome composition. We did not find a significant correlation between host phylogenetic distance and microbial dissimilarity, suggesting that any pattern of phylosymbiosis observed in bats is not driven by host evolution, but rather by ecological features. Investigation of microbial loads via culture-based approaches and quantitative PCR (qPCR) paired with observed spatial arrangements (e.g., via fluorescence *in situ* hybridization [68, 69]) throughout the gut and other anatomical sites of bats will provide important insights into the nature of bat-microbe associations and whether they are sustained, functional associations or transient encounters between bats and their environments. Analysis of morphological and physiological adaptations specific to flight in bats may also help elucidate why bats differ from nonflighted mammals with respect to the weak pattern of phylosymbiosis observed in this study.

## MATERIALS AND METHODS

### Field sampling and specimen preservation.

Sampling for this study was conducted at 20 field sites from the eastern coast of Kenya to the northern border of Uganda from August to October 2016 (Fig. 1; see also Tables S1 and S2 in the supplemental material). Nine families and 19 genera of bats (order Chiroptera) comprising 31 bat species and 497 individuals were collected as part of bird and small mammal biodiversity inventories. All sampling was conducted in accordance with the Field Museum of Natural History IACUC, and voucher specimens are accessioned at the Field Museum of Natural History (Table S2). Gut, skin, and oral samples were taken from each bat for microbial analyses. Gut samples consisted of fecal and gut lumen material collected directly from the freshly dissected distal end of the colon via scraping with sterilized tools; material was distributed evenly on Whatman FTA cards using sterile swab (for future studies, remaining intestinal material was flash-frozen whole in sterile cryogenic vials placed in liquid nitrogen). Studies comparing multiple sites throughout the chiropteran gastrointestinal tract have found no significant difference in bacterial community composition from different regions of the gastrointestinal tract (32); therefore, our sampling of distal colon should be representative of bacterial diversity throughout the intestine. For oral microbiome analyses, we preserved both buccal swabs in liquid nitrogen and tongue biopsy specimens in 95% ethanol (EtOH). Comparison of amplicon sequence variant (ASV) diversity obtained from paired subsets of each sample type revealed greater diversity recovered from tongue biopsy specimens (data not shown); tongues were therefore used for characterization of oral microbiomes in this study. Last, skin samples from five regions of the body (ear, wing membrane, tail membrane, chest, and back) were collected and pooled in 95% EtOH using sterile Integra Miltex 5-mm biopsy punches. The goal of sampling from five body regions was to maximize bacterial diversity recovered from the external skin surface of each individual. We based our storage medium selections on the recent study by Song et al. (70). Host age, sex, and mass were measured directly in the field following the capture and collection of bats. Dietary assignments (frugivorous or insectivorous) were made according to annotations from *Mammals of Africa* volume IV (71).

### Microbiome sequencing, characterization, and statistical analyses.

DNA extractions were performed on gut, tongue, and skin samples using the MoBio PowerSoil 96 well soil DNA isolation kit (catalog no. 12955-4; MoBio, Carlsbad, CA, USA) following the standard DNA extraction protocol outlined by the Earth Microbiome Project (http://www.earthmicrobiome.org/). We employed standardized PCR protocols (72–74) to amplify the V4 region of the 16S rRNA gene, using the 515f and 806r primers and mitochondrial blockers to reduce amplification of host mitochondrial DNA. Sequencing was performed using paired-end 150-base reads on an Illumina HiSeq sequencing platform through the University of Illinois at Chicago Sequencing Core, following standardized sequencing protocols described by Caporaso et al. (73). Full DNA extraction, amplification, and sequencing protocols and standards are available at http://www.earthmicrobiome.org/protocols-and-standards.

Following standard demultiplexing and quality filtering using the Quantitative Insights Into Microbial Ecology pipeline (QIIME2) (75) and vsearch8.1 (76), ASVs were identified using the Deblur method (35) and taxonomy was assigned using the Greengenes Database (May 2013 release; http://greengenes.lbl.gov), which was sufficiently able to classify the majority of reads to bacterial taxa (4.1% reads were unclassified). Analyses and statistical tests were performed on nonrarefied data consisting of libraries containing >1,000 reads and transformed to library read depth (transformed as amplicon read count/Σ total reads for each library). According to a recent study by McMurdie and Holmes (77), rarefying 16S data is inappropriate for the detection of differentially abundant species. However, for the purposes of comparison, we compared both libraries rarefied to a read depth of 10,000 reads and libraries filtered to those containing >1,000 reads (negative controls all contained fewer than 1,000 reads and were filtered at this step). Analyses of α- and β-diversity produced statistically similar results, with no significant differences observed between the rarefied and nonrarefied data. We thus chose to report results of nonrarefied data, based on these observations and the recommendation of McMurdie and Holmes (77). Following filtering, data were divided into subsets for analyses according to sample type, host genus, and locality (or some combination thereof). Site-specific analyses were performed only for sites from which five or more individual bats were sampled. We calculated α-diversity for each sample type (gut, oral, and skin) using the Shannon index and measured species richness based on the number of observed ASVs. Significance of differing mean values for each diversity calculation was determined using the Kruskal-Wallis rank sum test, followed by a *posthoc* Dunn test with Bonferroni-corrected *P* values. Weighted UniFrac β-diversity was calculated using the relative abundance of each ASV (calculated as ASV read depth over total read depth per library). To measure the influence of host taxonomy and host traits on unweighted and weighted UniFrac distances, we used the adonis2 function (R package vegan2.4-2) (78) to perform permutational multivariate analyses of variance (PERMANOVA) with 1,000 permutations. The nested nature of host taxonomy was taken into account, testing first host family, then host genus nested within family, then host species nested within genus nested within family. For analysis of locality, we examined the marginal effects of both host locality and host species (Table 2, formula 1). Diet was assessed independently of all other variables while setting locality as strata (pseudo-F permutations constrained within individual sampling localities) to account for the effect of locality (Table 2, formula 2). For analysis of the effects of host age, sex, and mass, we examined the marginal effects of each variable while setting host species as strata (pseudo-F permutations constrained within individual host species) to account for the effect of host species (Table 2, formula 3), and *P* values were adjusted for multiple comparisons using the false-discovery rate. To assess the extent to which uneven sampling may have driven results, we performed additional PERMANOVA on reduced data sets that included only species for which we sampled ≥10 and ≥20 individuals. Analysis of composition of microbiomes (ANCOM) was used to identify bacterial ASVs that were differentially abundant between bat dietary groupings (frugivorous and insectivorous), using a *P* value cutoff of <0.05 with Benjamini-Hochberg correction (79). Additional R packages used for analyses and figure generation included ggplot2 (80) and dplyr (81).

### Bat phylogenetic reconstruction and Mantel’s test.

DNA was extracted from pectoral muscle tissue specimens from bats and sequenced for mitochondrial cytochrome *b* (cyt *b*) using the primer pair LGL 765F and LGL 766R to amplify the entire cyt *b* gene (82, 83) for 28 bat species and Mus musculus as an outgroup. PCR amplification and sequencing were conducted as described by Demos et al. (84). The best-supported model of nucleotide substitution for cyt *b* was determined using the Bayesian information criterion (BIC) on the maximum-likelihood topology estimated independently for each model in jMODELTEST2 (85) on CIPRES Science Gateway v.3.1 (86). Bayesian estimates of cyt *b* gene trees were made using the program BEAST v.2.5.1 (87) on the CIPRES portal. Four independent Markov chain Monte Carlo (MCMC) runs of 4 × 10^8^ generations were carried out using a generalized time-reversible GTR+I+Γ substitution model, a log normal relaxed-clock model, and the Yule tree prior. We used six fossil calibrations to estimate the ages of nodes in the phylogeny based on parameters from the following extinct taxa from Amador et al. (36): *Cuvieramops*, *Khonsunycteris*, *Mormopterus*, *Onychonycteris*, *Rousettus*, and *Tachypteron*. The minimum age of nodes was determined using the log normal distribution. Values for the mean and standard deviation of fossil ages are from Amador et al. (36) with the age of the fossil as “offset.” We used Tracer v.1.7 to assess convergence and stationarity of model parameters based on trace files and effective sample size (ESS) values.

All newly generated sequences are available on GenBank (MN064727 to MN064748). Once generated, the BEAST chronogram was used to produce a matrix of pairwise phylogenetic distances between each bat species. To evaluate the effect of host phylogeny on microbiome dissimilarity (i.e., phylosymbiosis), we performed Mantel tests (10,000 permutations) between this phylogenetic distance matrix and matrices of unweighted and weighted UniFrac dissimilarity. UniFrac dissimilarity values were produced by measuring all pairwise dissimilarities between samples and then grouping samples by species and calculating the mean dissimilarities between each species pair (i.e., mean pairwise dissimilarities between all possible host species pairs).

### Machine learning.

A supervised machine learning approach was used to produce random forests (RFs) for the classification of different variables. RFs were constructed using 1,000 decision trees and subsets of ASV data via the supervised_learning.py script implemented in QIIME (75), which utilized 80% of each input data set to train classification models, and tested the accuracy of the models on the remaining 20% of data, with 1,000 bootstrap replicates. We tested the ability of RFs to accurately classify gut, oral, and skin microbiota by host taxon at the family, genus, and species levels, sampling locality, and dietary niche (frugivore or insectivore). For a detailed explanation on the application of random forests and machine learning to 16S rRNA microbiome data, see Knights et al. (88).

### Data availability.

For a complete list of packages and code for microbiome analyses, see http://github.com/hollylutz/BatMP. All 16S rRNA sequence and sample metadata are publicly available via the QIITA platform under study identifier (ID) 11815 and the European Bioinformatics Institute (EBI) under accession number PRJEB32520. Host sequence data are available via NCBI under GenBank accession numbers MN064727 to MN064748.
