# Supplementary figures and images for: Ecology and Host Identity Outweigh Evolutionary History in Shaping the Bat Microbiome
Source: mSystems. 2019 Nov 12;4(6):e00511-19. doi: 10.1128/mSystems.00511-19 (PMC7407897; doi:10.1128/mSystems.00511-19)

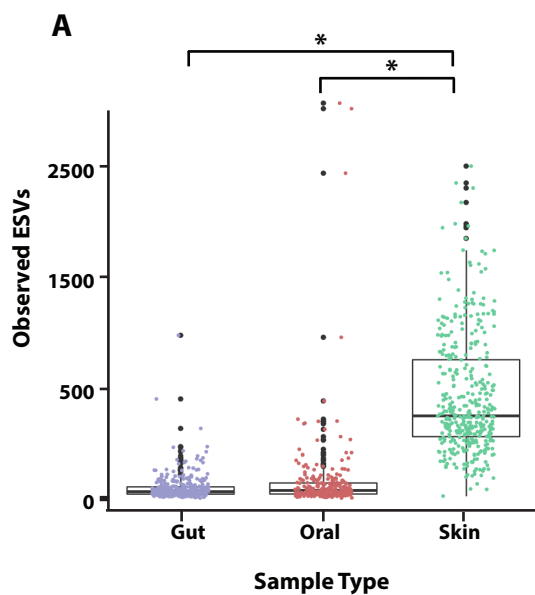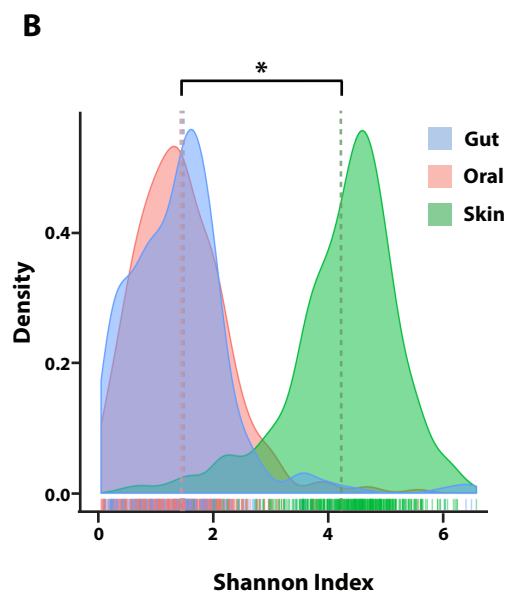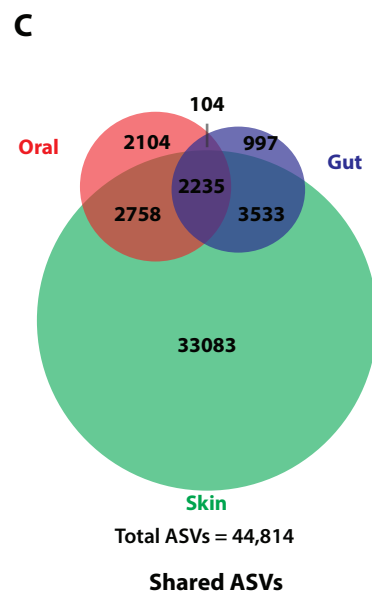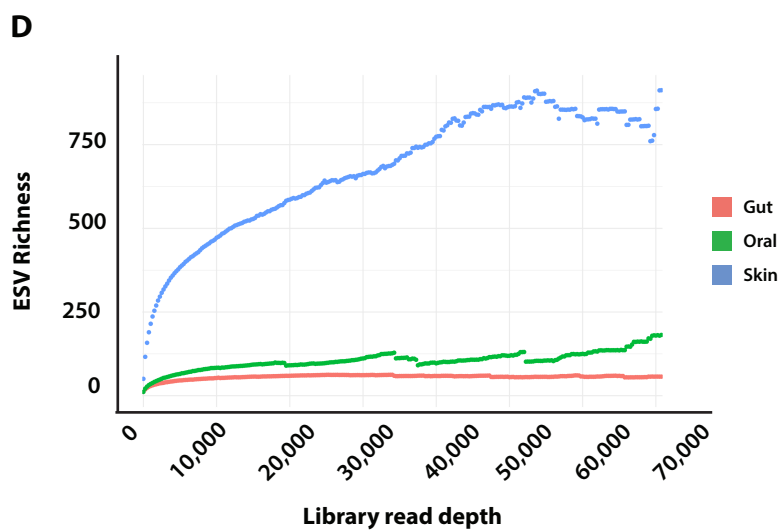

Supplement: FIG S1 [file mSystems.00511-19-sf001.pdf]

**A**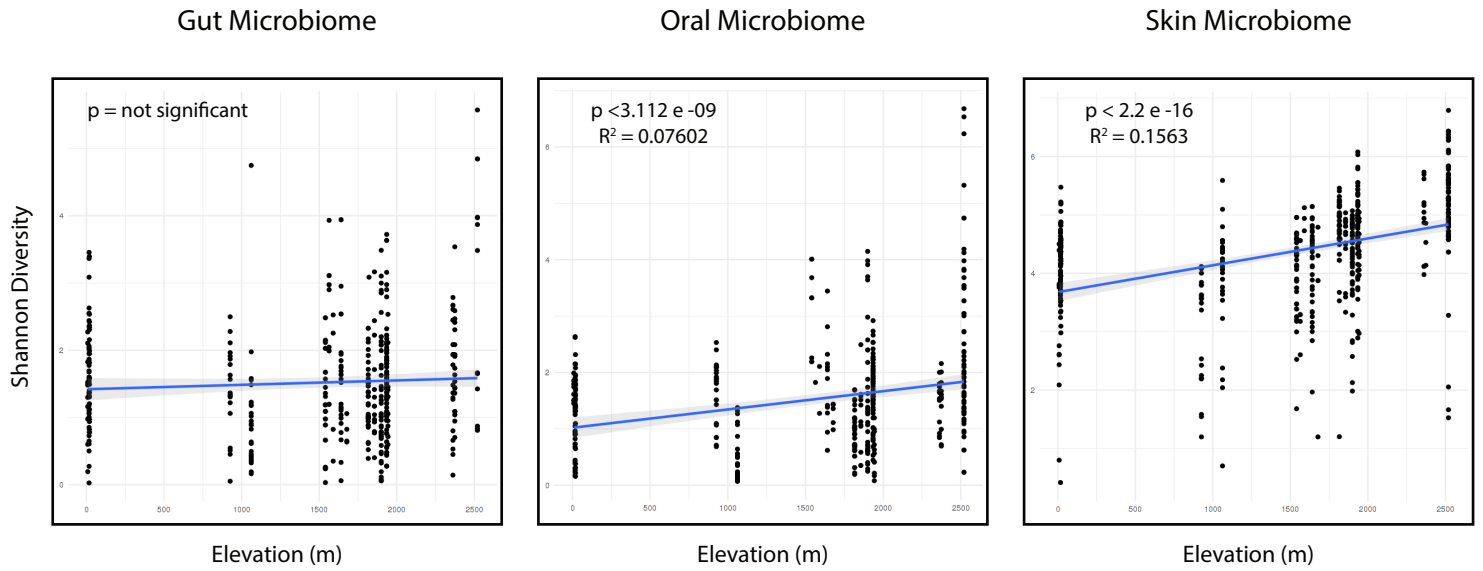**B**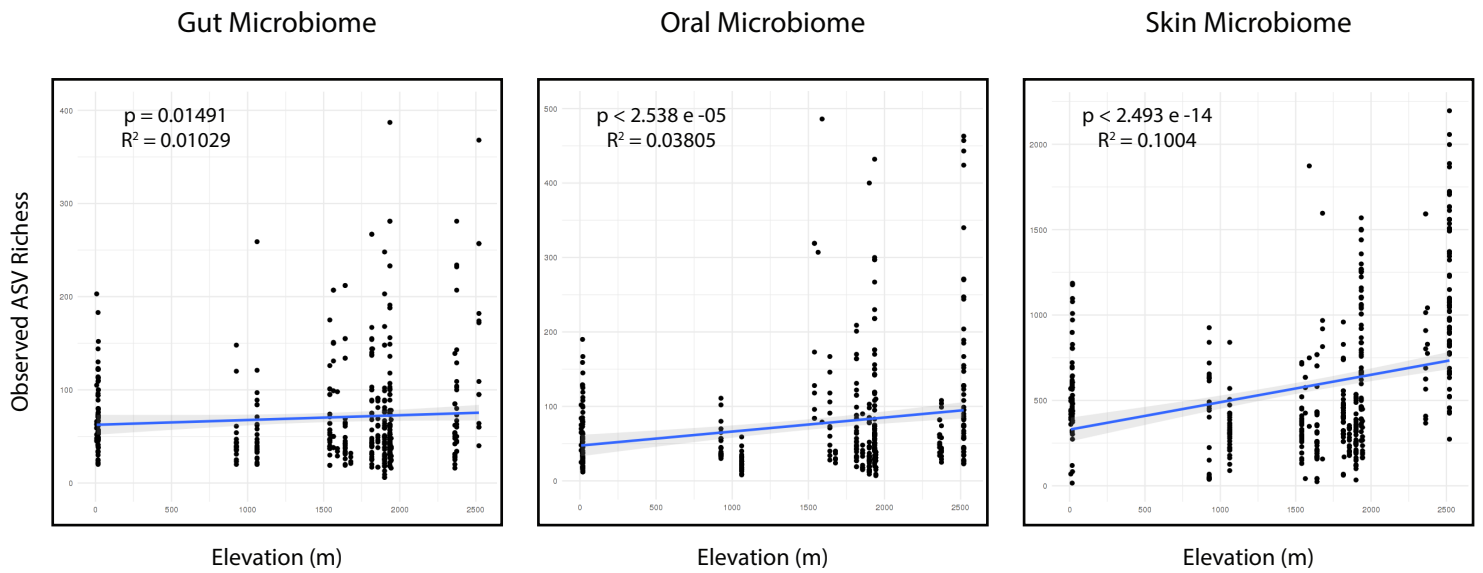

Supplement: FIG S2 [file mSystems.00511-19-sf002.pdf]

A

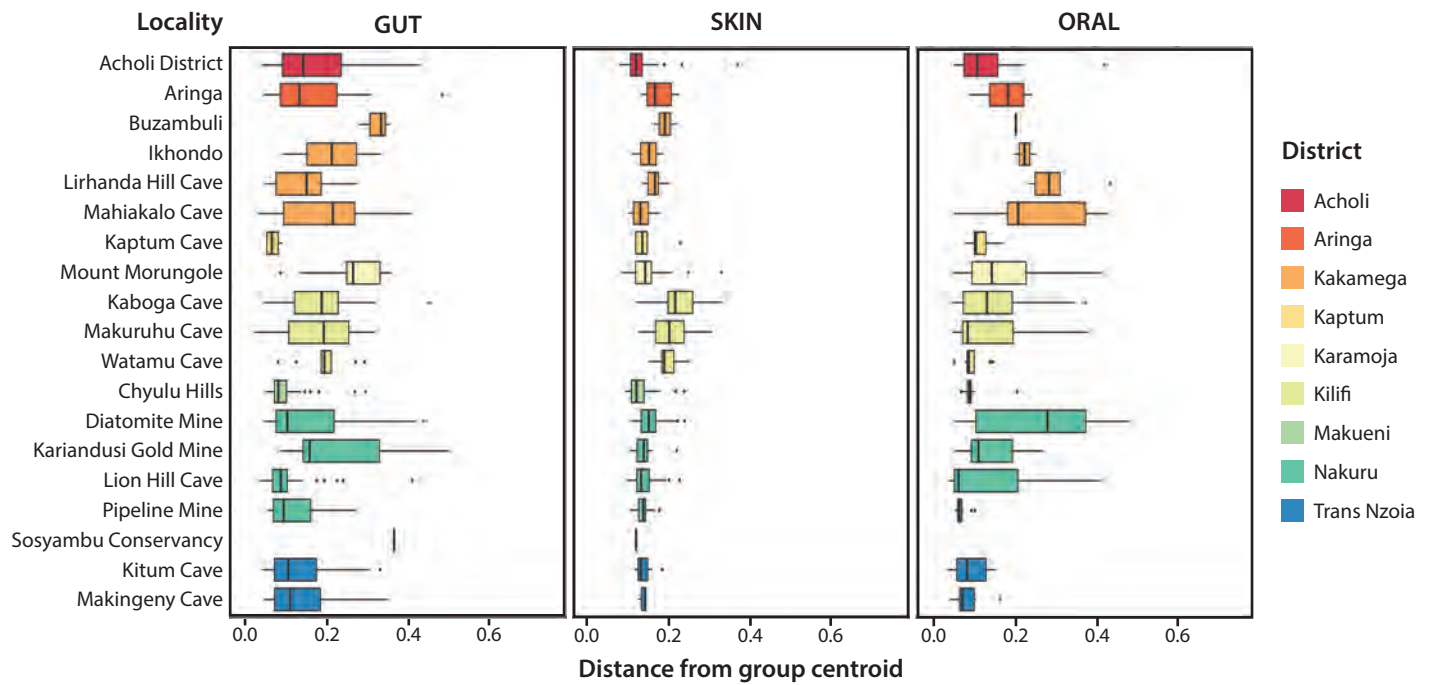

B

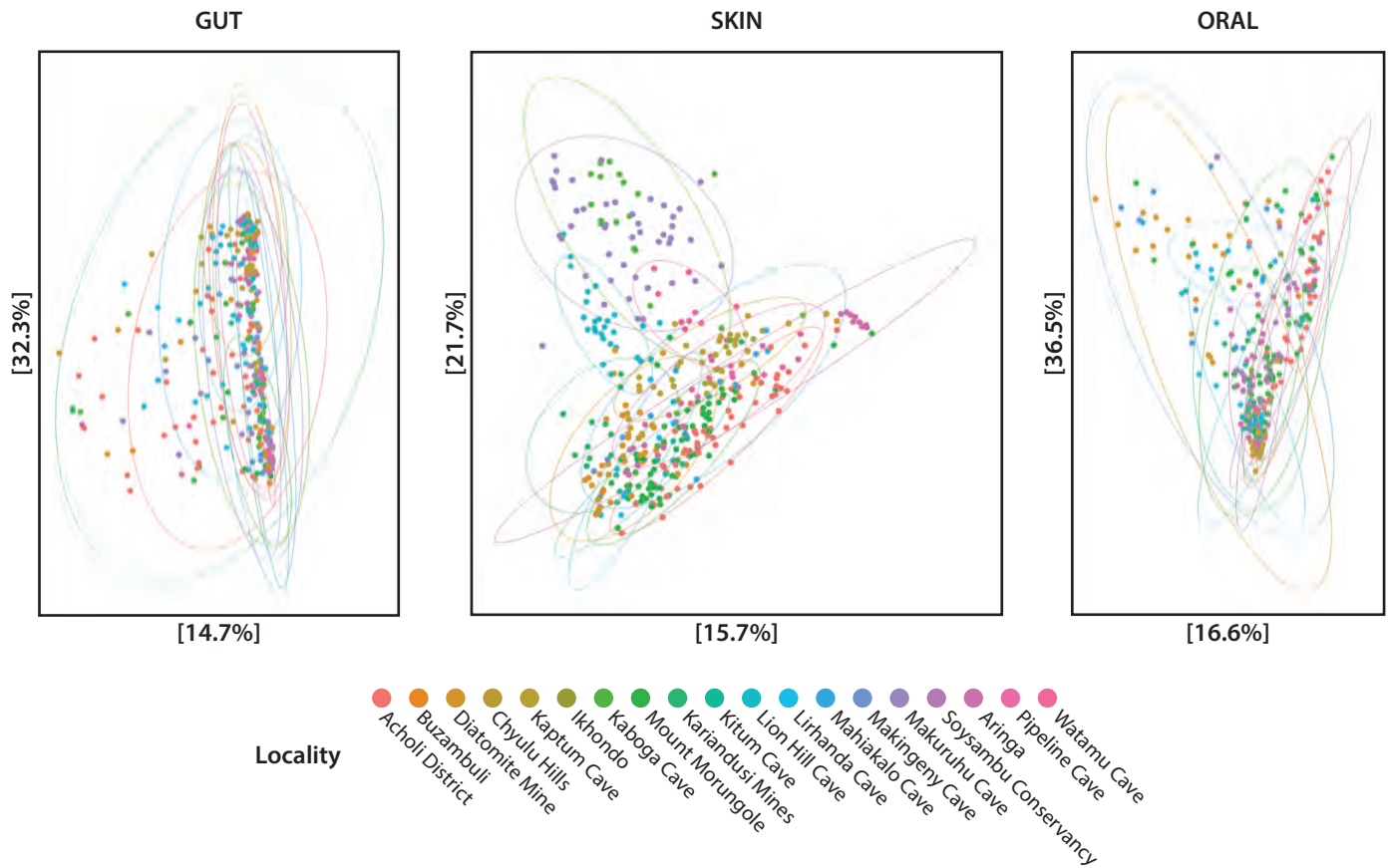

Supplement: FIG S3 [file mSystems.00511-19-sf003.pdf]

**A**

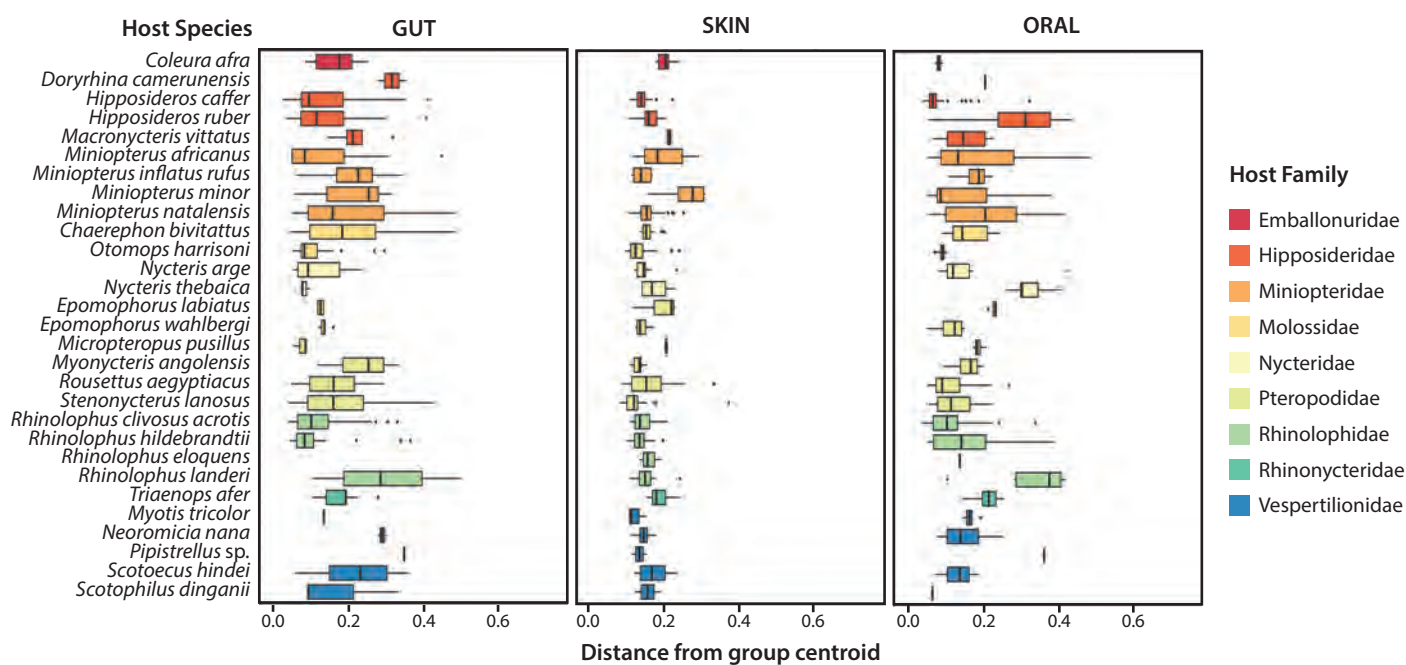

## B

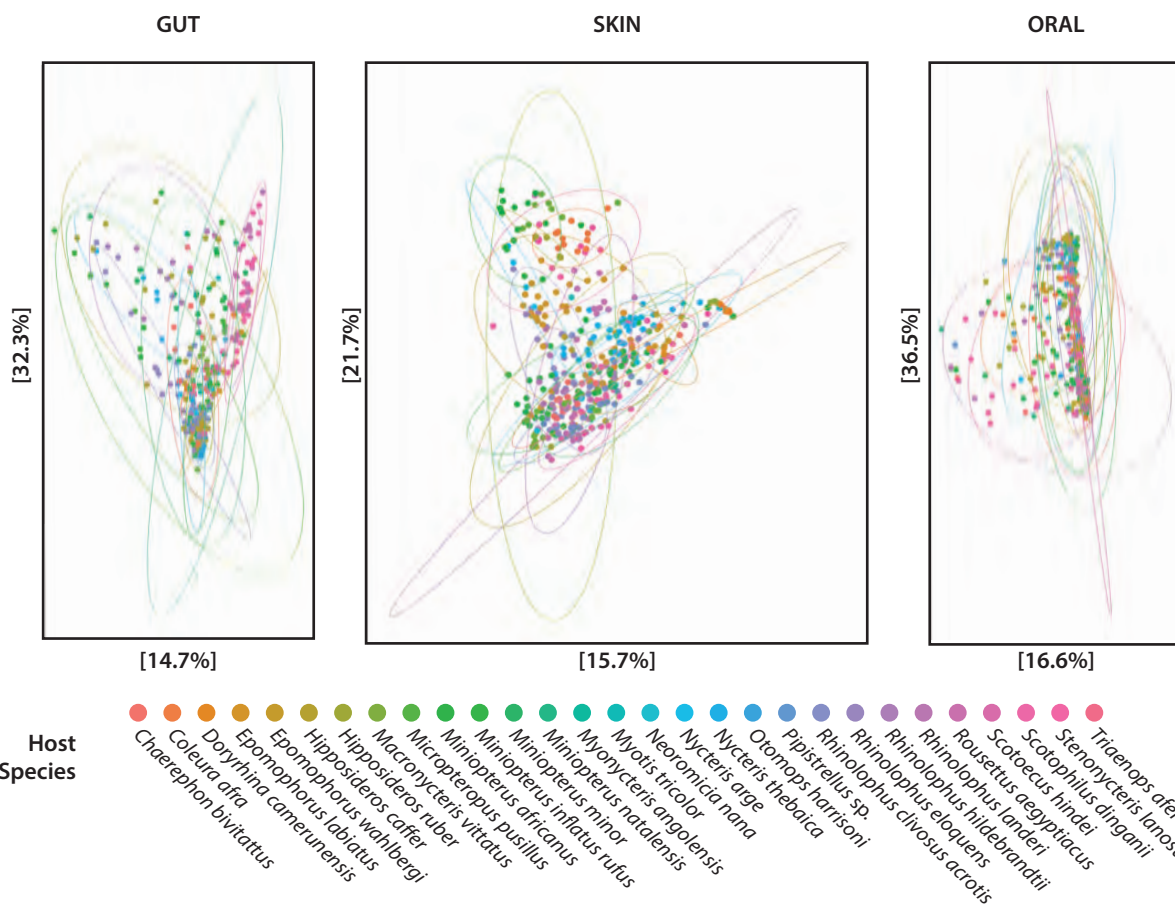

Supplement: FIG S4 [file mSystems.00511-19-sf004.pdf]

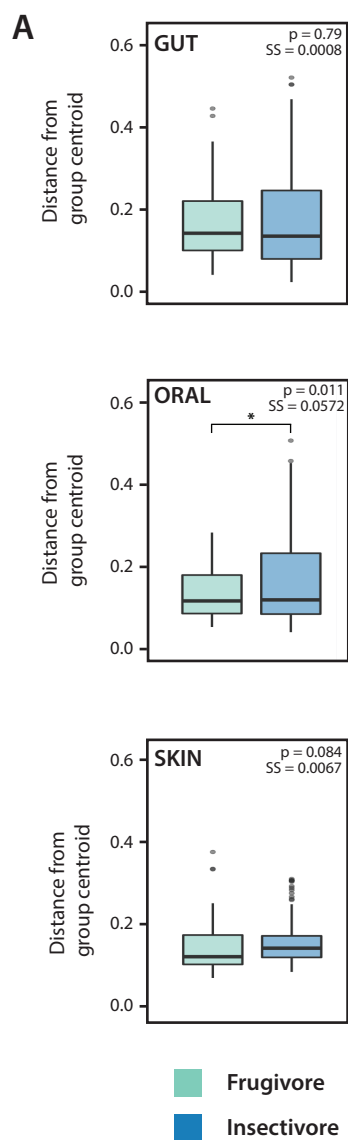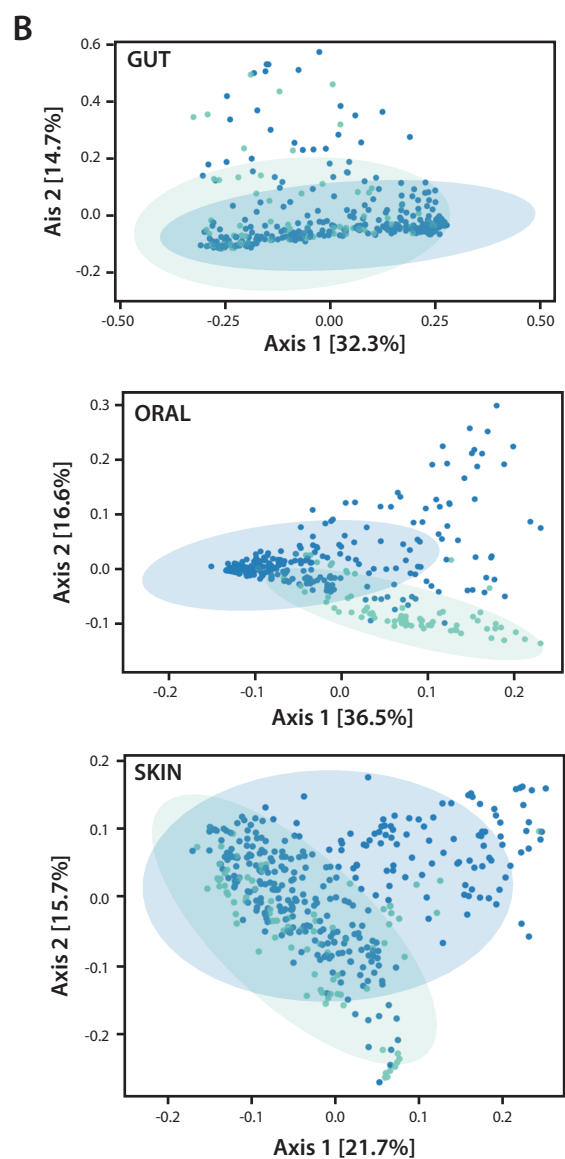

Supplement: FIG S5 [file mSystems.00511-19-sf005.pdf]
